# Non-invasive Adenocarcinoma of the Vermiform Appendix: Incidence and Report of Four Cases among 512 Appendectomies

**DOI:** 10.4021/gr2009.01.1267

**Published:** 2009-07-20

**Authors:** Tadashi Terada

**Affiliations:** Department of Pathology, Shizuoka City Shimizu Hospital, Miyakami 1231 Shimizu-Ku, Shizuoka 424-8636, Japan. Email piyo0111jp@yahoo.co.jp

**Keywords:** Appendix, Adenocarcinoma, Non-invasive carcinoma, appendicitis, Histopathology

## Abstract

Tumors of the vermiform appendix are relatively rare. More than 50% of appendiceal tumors are carcinoid tumors. The author reviewed 512 consecutive pathological specimens of appendectomies in last ten years in our pathology laboratory in search for appendiceal tumors. As the results, 4 cases (incidence: 0.8%) of non-invasive adenocarcinoma were found. No other tumors including carcinoid tumors were recognized. The age of the 4 patients with adenocarcinoma was 48, 39, 84 and 86 years, respectively. Male to female ratio was 3:1. The clinical diagnoses were acute appendicitis in 2 cases and suspected malignancy in 2 cases. The post-operative outcome was good without metastasis, recurrence, and pseudomyxoma peritonei. Pathologically, all the 4 tumors were non-invasive adenocarcinomas: 2 cases were flat type adenocarcinoma, 1 case was papillary adenocarcinoma, and 1 case was mucinous adenocarcinoma. Immunohistochemically, expression of p53 protein was observed in all the 4 cases, and Ki-67 labeling ranged from 40% to 90%. The results suggest that incidence of appendiceal adenocarcinoma was 0.8% of all appendectomies, and that non-invasive adenocarcinoma of the appendix shows variable morphologies, and that postoperative clinical outcome of non-invasive appendiceal tumor is good.

## Introduction

Tumors of vermiform appendix are relatively rare conditions. More than 50% of appendiceal tumors are carcinoid tumor [1]. Adenocarcinoma of the appendix accounts for 58% of malignant appendiceal tumors [2]. The incidence of adenocarcinoma is reported to be 0.1% [2]. According to WHO, adenocarcinoma of the appendix is defined as a malignant epithelial neoplasm of the appendix with invasion beyond the muscularis mucosa [2]. The appendiceal carcinomas are classified into adenocarcinoma, mucinous adenocarcinoma, singet-ring cell carcinoma, small cell carcinoma, and undifferentiated carcinoma [2]. There are several comprehensive studies of appendiceal carcinoma [3-10]. The author reviewed 512 consecutive pathological specimens of appendectomies in last ten years in our pathology laboratory in search for appendiceal tumors. The author herein reports the results.

## Case reports

The author reviewed 512 consecutive pathological specimens of appendectomies in last ten years in our pathology laboratory in search for appendiceal tumors. Clinical records were also reviewed. In carcinoma cases, an immunohistochemical study was performed, using Dako’s Envision methods, (Dako Corp. Glostrup, Denmark), as previously reported [11, 12]. The antibodies used were anti-p53 protein (DO-7, Dako) and anti Ki-67 antigen (MIB-1, Dako).

Among the 512 appendiceal specimens, 4 cases of non-invasive adenocarcinoma were identified. Therefore, the incidence of appendiceal adenocarcinoma was 0.8% of all appendectomies. No cases of other tumors including carcinoid tumors were found.

### Case 1

**Figure 1:**
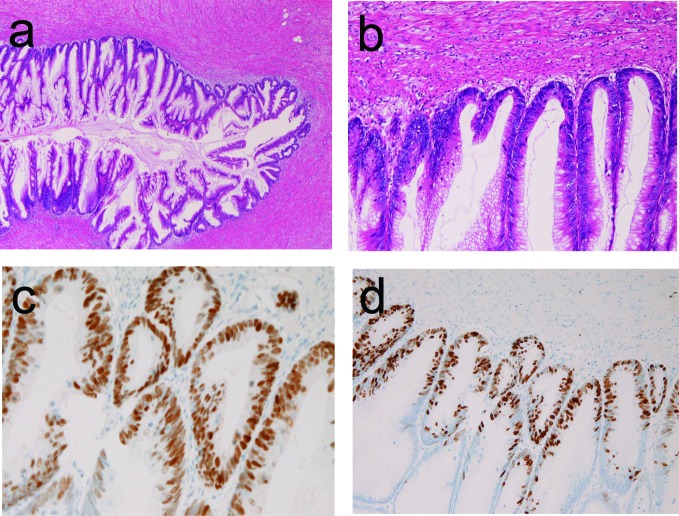
(a) Low power view of papillary adenocarcinoma of the appendix in case 1. Papillary proliferation is apparent. No invasion is seen. HE, x 20. (b) Higher power view of Figure 1A. The cellular atypia is evident. HE, x 200. (c) The tumor cells are positive for p53 protein, Immunostaining, x 200. (d) The Ki-67 labeling is 90%. Immunostaining, x 100.

A 48-year-old woman was admitted to our hospital because of acute right lower abdominal pain. Clinically, acute appendicitis was diagnosed, and an appendectomy was performed. Pathologically, the appendix was small and fibrotic (Fig. 1a). Papillary epithelial proliferation was recognized in the appendiceal mucosa (Fig. 1a). The tumor epithelium showed cellular atypia regarded as malignant (Fig. 1b). No invasive features were recognized (Fig. 1a). Immunohistochemically, p53 protein was positive (Fig. 1c) and Ki-67 labeling was 90% (Fig. 1d). No pseudomyxoma peritonei was recognized. The patient is healthy without metastasis and recurrence 37 months after the operation.

### Case 2

**Figure 2:**
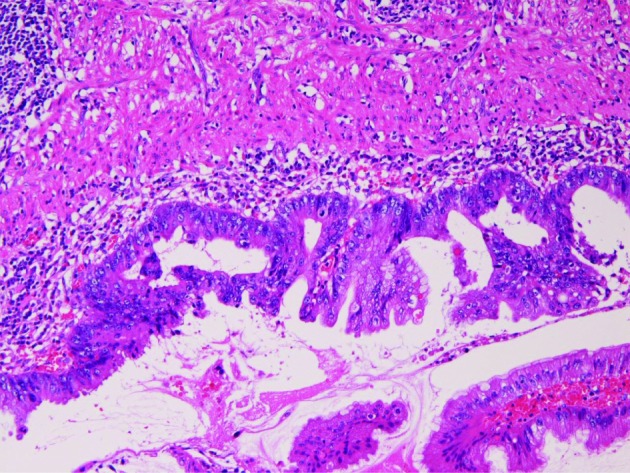
Flat type adenocarcinoma in case 2. HE, x 200.

An 84-year-old man was admitted to our hospital complaining of acute abdominal pain. Clinically, acute appendicitis was diagnosed, and an appendectomy was performed. Pathologically, the appendix showed acute phlegmonous appendicitis. Flat type adenocarcinoma was recognized in the mucosa (Fig. 2). The carcinoma cells showed enough cellular atypia regarded as adenocarcinoma. No invasive features were recognized. P53 protein was positive and Ki-67 labeling was 40%. The patient is healthy without metastasis and recurrence 51 months after operation.

### Case 3

**Figure 3:**
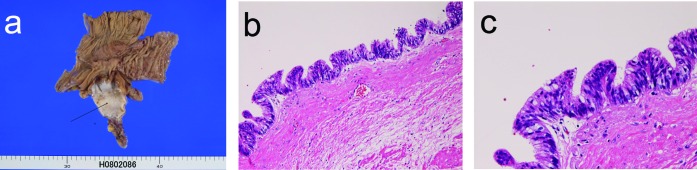
(a) The resected appendix shows cystic dilation (arrow) in case 3. (b) The cystic lining is adenocarcinoma cells. HE, x 100. (c) The cellular atypia is enough to be regarded as adenocarcinoma. HE, x 200.

A 39-year-old man presented with chronic abdominal pain and fever. Imaging modalities including US, CT and MRI revealed cystic dilation of the appendix. Resection of appendix, terminal ileum and cecum was performed (Fig. 3a) under the clinical diagnosis of suspected appendiceal tumor. Grossly, the proximal appendix showed cystic dilation (Fig. 3a). Histologically, the cystic dilation was covered by flat type atypical epithelium with cellular atypia (Fig. 3B). The atypia was enough to be diagnosed as adenocarcinoma (Fig. 3c). No invasive features were recognized (Fig. 3b, c). P53 protein was positive and Ki-67 labeling was 50%. The patient is healthy without metastasis and recurrence 8 months after the operation.

### Case 4

**Figure 4:**
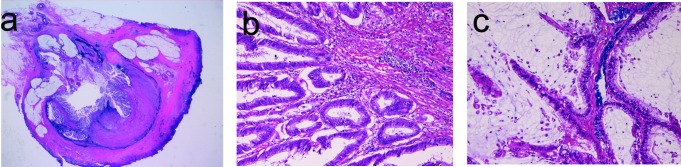
(a) Loupe figures of appendiceal mucinous adenocarcinoma in case 4. Papillary epithelial proliferation and intraluminal mucus are evident. No invasion of tumor cells is recognized. (b) Higher power view of the mucosa of the appendix. Papillary adenocarcinoma is evident. HE, x 200. (c) The intraluminal area show adenocarcinoma cells and much mucus. HE, x 200.

An 86-year-old woman was admitted to our hospital because of acute chronic abdominal pain. Colon endoscopy revealed a tumor at the orifice of the appendectomy, and biopsies from the tumor showed atypical cells suggestive of adenocarcinoma. Therefore, resection of appendix, terminal ileum and cecum was performed under the clinical diagnosis of probable appendiceal adenocarcinoma. Grossly, the appendiceal lumen was filled with papillary epithelial proliferation and much mucus (Fig. 4a). Histologically, the papillary epithelial proliferation consisted of atypical cells regarded as adenocarcinoma (Fig. 4b). Much mucus was impacted in the lumen (Fig. 4c). No invasion was recognized (Fig. 4a, b). The diagnosis was mucinous adenocarcinoma. Immunohistochemically, p53 protein was positive and Ki-67 labeling was 60%. No pseudomyxoma peritonei was recognized. The patient is healthy without metastasis and recurrence 7 years after the operation.

## Discussion

The most common appendiceal tumor is carcinoid tumor, followed by carcinoma [1, 2]. The present series did not contain carcinoid tumors, although 4 cases of adenocarcinoma were identified. These findings suggest that appendiceal carcinoid tumors and benign epithelial tumors are infrequent in our hospital.

In the present series, the incidence of adenocarcinoma was 0.8 % of all appendectomies. In the WHO blue book [2], the incidence of adenocarcinoma is 0.1% of all appendectomies. According to the data of Marudanayagam et al [3], the incidence of carcinoid tumor, adenocarcinoma and mucinous cystadema was 0.52%, 0.39% and 0.6% of all appendectomies, respectively. The incidence of 0.8% of the present series is highest. These findings suggest that appendiceal adenocarcinoma is more prevalent in our hospital.

Clinically, two cases (case 1 and case 2) of the present series showed clinical features of acute appendicitis. The other two cases (case 3 and case 4) in the present cases showed some clinical features of appendiceal tumors. In particular, imaging modalities identified abnormities of the appendix in these two cases. These findings suggest that clinicians should be aware of appendiceal carcinoma even in patients with typical clinical features of acute appendicitis. Further, imaging techniques including US, CT and MRI are essential for identification of appendiceal tumors. Pathologists also should carefully examine the appendectomies. The prognosis was good in the present series.

Pathologically, the appendiceal adenocarcinoma of the present series was papillary (or villous) adenocarcinoma in one case, flat type adenocarcinoma in two cases, and mucinous adenocarcinoma in one case. All the four cases were in situ adenocarcinomas without apparent invasion. These findings may indicate that the present adenocarcinomas were in early stages in the carcinomatous progression. No pseudomyxoma peritonei [8-10] was noted in the present cases, suggesting the above hypothesis. The mucinous adenocarcinoma in case 4 of the present series is classified as low-grade mucinous adenocarcinoma, according to Misdraji et al [6].

In the present series, immunoreactive p53 protein was expressed in all the 4 adenocarcinomas. Kabbani et al [7] suggested that p53 expression was found in only 1 (3%) case of the 30 appendiceal mucinous adenocarcinoma. In contrast, Yajima et al [5] showed p53 positive cells percentage was 29 % in appendiceal mucinous adenocarcinoma. Much more studies are required as to p53 gene status in appendiceal adenocarcinoma. In the present series, Ki-67 labeling ranged from 40 % to 90 %, indicating a high proliferative activity of tumor cells.

In summary, the present series suggest that incidence of appendiceal tumor was 0.8% of all appendectomies. All the detected 4 adenocarcinomas were non-invasive adenocarcinomas of the appendix showing variable morphologies.
